# Cortico-Amygdala Coupling as a Marker of Early Relapse Risk in Cocaine-Addicted Individuals

**DOI:** 10.3389/fpsyt.2014.00016

**Published:** 2014-02-27

**Authors:** Meredith J. McHugh, Catherine H. Demers, Betty Jo Salmeron, Michael D. Devous, Elliot A. Stein, Bryon Adinoff

**Affiliations:** ^1^Neuroimaging Research Branch, Intramural Research Program, National Institute on Drug Abuse, Baltimore, MD, USA; ^2^Avid Radiopharmaceuticals, Philadelphia, PA, USA; ^3^VA North Texas Health Care System, Dallas, TX, USA; ^4^Department of Psychiatry, University of Texas Southwestern Medical Center, Dallas, TX, USA

**Keywords:** cocaine, relapse, addiction, amygdala, connectivity, neuroimaging, ventromedial, prefrontal

## Abstract

Addiction to cocaine is a chronic condition characterized by high rates of early relapse. This study builds on efforts to identify neural markers of relapse risk by studying resting-state functional connectivity (rsFC) in neural circuits arising from the amygdala, a brain region implicated in relapse-related processes including craving and reactivity to stress following acute and protracted withdrawal from cocaine. Whole-brain resting-state functional magnetic resonance imaging connectivity (6 min) was assessed in 45 cocaine-addicted individuals and 22 healthy controls. Cocaine-addicted individuals completed scans in the final week of a residential treatment episode. To approximate preclinical models of relapse-related circuitry, separate seeds were derived for the left and right basolateral (BLA) and corticomedial (CMA) amygdala. Participants also completed the Iowa Gambling Task, Wisconsin Card Sorting Test, Cocaine Craving Questionnaire, Obsessive-Compulsive Cocaine Use Scale and Personality Inventory. Relapse within the first 30 days post-treatment (*n* = 24) was associated with reduced rsFC between the left CMA and ventromedial prefrontal cortex/rostral anterior cingulate cortex (vmPFC/rACC) relative to cocaine-addicted individuals who remained abstinent (non-relapse, *n* = 21). Non-relapse participants evidenced reduced rsFC between the bilateral BLA and visual processing regions (lingual gyrus/cuneus) compared to controls and relapsed participants. Early relapse was associated with fewer years of education but unrelated to trait reactivity to stress, neurocognitive and clinical characteristics or cocaine use history. Findings suggest that rsFC within neural circuits implicated in preclinical models of relapse may provide a promising marker of relapse risk in cocaine-addicted individuals. Future efforts to replicate the current findings and alter connectivity within these circuits may yield novel interventions and improve treatment outcomes.

## Introduction

Addiction to cocaine is a debilitating condition characterized by poor treatment retention and high relapse rates (1, 2). Identifying factors that drive relapse following abstinence from cocaine remains one of the major challenges for clinicians and researchers alike (2–4). Evidence that cocaine addiction is associated with marked neurobiological changes, or features that either result from or predispose an individual to compulsive cocaine use (5), has led to the emergence of human neuroimaging as a potential tool for identifying neural markers of relapse risk and treatment efficacy. The present study builds on this effort, addressing neural circuits arising from the amygdala, a brain region heavily implicated in cocaine addiction, withdrawal and relapse.

The amygdala and its associated circuitry have been implicated in various relapse-related processes including craving, anxiety, and reactivity to stress following both acute and protracted withdrawal from cocaine and other drugs of abuse (6–9). In cocaine-addicted individuals, drug-associated cues and drug craving elicit amygdala activation coupled with engagement of a distributed network of cortical and striatal regions implicated in relapse (10–13). Cocaine-addicted individuals also show disrupted amygdala coupling with prefrontal regions during the down-regulation of negative affect (14) and a negative relationship between amygdala activation to reward anticipation and retention in treatment (15). Although imaging studies suggest a role for the amygdala in cocaine-addiction and relapse-related processes, they have traditionally treated the amygdala as a single, functionally homogeneous structure instead of a heterogeneous complex of nuclei (16). Based on shared cytoarchitectonic features and circuitry, the nuclei of the amygdala have been grouped into basolateral and corticomedial divisions (16–18) which, importantly, have been shown to mediate dissociable processes in addiction and relapse (19).

The basolateral division of the amygdala (BLA) consists of the basal, lateral, and accessory basal nuclei (16, 20). Traditionally considered the primary input zone for the amygdala, the BLA is extensively connected with the sensory thalamus and sensory association cortices as well as medial temporal regions involved in memory (16, 20, 21). The BLA in turn projects back to medial temporal regions and prefrontal cortices, as well as the dorsal and ventral striatum (20). Rodent models of cocaine-addiction and relapse have implicated the BLA in the consolidation and reconsolidation of cocaine-cue and cocaine-context associations that drive cocaine-seeking once established, as well as the behavioral expression of these associations (7, 22–26). For example, the ability of discrete cocaine-associated cues and cocaine-associated contexts to reinstate extinguished cocaine-seeking is blocked by lesions to the BLA (7, 8, 27).

The remaining core nuclei of the amygdala (cortical, medial, and central nuclei) form the corticomedial division (CMA) (16). Although the CMA receives some sensory input, it also functions as the primary functional output for the amygdala via projections to the cortex, hypothalamus, midbrain and brainstem including ascending cholinergic and monoaminergic systems (16, 18, 20). The CMA, in particular the central nucleus (CeA), has been implicated in the incubation of cocaine craving, a phenomena characterized by sensitization to cocaine-associated cues (i.e., enhanced cue-induced reinstatement of cocaine-seeking) that increases with time across the first few months following withdrawal from cocaine self-administration (28, 29). The preclinical incubation phenomena mirrors the change in craving observed in cocaine-addicted individuals with time since last cocaine use (30). The CeA mediated mechanism implicated in the incubation of craving involves glutamatergic (excitatory) input to the CeA, which mediates protein synthesis processes involved in learning and memory (31, 32).

The CeA is also the locus of neuroadaptations that accompany acute and protracted withdrawal from drugs of abuse (26, 33). During acute withdrawal, neuroadaptations in the CeA, such as increased extracellular levels of the stress-related neuropeptide corticotrophin releasing factor (CRF), have been shown to mediate enhanced baseline anxiety and increased withdrawal-induced drug-seeking (34, 35). The CeA is also a critical part of the circuitry that mediates stress-induced reinstatement of cocaine-seeking (36) and has been implicated in the progressive elevation in reactivity to stress, which occurs over longer-term abstinence and may underlie vulnerability to relapse in response to stressful life events (9, 37).

Both amygdala divisions are reciprocally interconnected with the orbitofrontal cortex (OFC) as well as medial prefrontal, dorsolateral, and anterior cingulate cortices (20, 21, 38–40). Prefrontal-amygdala circuits have been implicated in both facilitating and inhibiting drug-seeking behavior (41). For example, coupling between the BLA and the prelimbic cortex – the rodent homolog of the dorsal anterior cingulate/dorsomedial prefrontal cortex (dACC/dmPFC) in humans – facilitates cocaine-seeking behavior, in particular reinstatement of extinguished cocaine-seeking (42, 43). The infralimbic cortex, which is the rodent homolog of the human rostral anterior cingulate/ventromedial prefrontal cortex (rACC/vmPFC), is instead implicated in inhibiting and extinguishing cocaine-seeking (41, 44, 45). Cocaine-addicted individuals also evidence diminished gray matter volume in the vmPFC (46) as well as deficits in decision making that resemble individuals with damage to this region (47). Extant human and animal literature also directly links the rACC/vmPFC to down regulation of amygdala output (41, 48–53) and a consequent reduction in negative affect (41, 48, 49, 51). In rodents, it is likely that this mechanism involves glutamatergic projections from the infralimbic cortex, which synapse on GABAergic (inhibitory) neurons in and around the CeA, resulting in inhibition of CeA output (41).

In sum, preclinical research suggests that BLA and CMA circuits may play critical and dissociable roles in relapse to cocaine use following treatment. In the present study, we employed resting-state functional connectivity (rsFC) to study the contribution of these circuits to relapse risk in a sample of treatment-seeking cocaine-addicted individuals. rsFC measures covariation in spontaneous low frequency (0.01–0.1 Hz) fluctuations of the blood-oxygen-level-dependent (BOLD) functional magnetic resonance imaging (fMRI) signal between different brain regions (54). Task-based neuroimaging analyses rely on relatively small changes in neuronal metabolism for their signal (at most 5%) and have traditionally treated ongoing spontaneous activity, which forms the basis for rsFC analyses, as noise (55). Critically, when assessed across the whole-brain, spatio-temporal patterns of rsFC emerge that correspond to known functional networks (54, 56) and vary as a function of disease states such as addiction (57). Further, evidence strongly suggests that rsFC strength predicts subsequent task-based behavioral performance and BOLD activation (58–61). Other advantages of rsFC over more traditional task-based approaches include an enhanced signal to noise ratio, simultaneous measurement of multiple cortical systems, and avoiding the limitations inherent to task-based studies such as practice effects or discerning activation differences related to behavioral performance or strategy from underlying disease-related variance in brain function (55).

In an earlier study and a unique population to the present study, we found that cocaine-addicted individuals evidenced reduced rsFC between the amygdala and vmPFC (62), a circuit which, as described above, may be involved in inhibition/extinction of cocaine-seeking behavior (41). While this and studies cited above suggest that dysregulation in amygdala circuits may contribute to cocaine-addiction in humans, the role of amygdala circuitry in relapse risk has not been addressed. Moreover, all previous neuroimaging studies of cocaine addiction have treated the amygdala as a single structure, overlooking important functional dissociations as outlined above between BLA and CMA divisions. Distinguishing these divisions would also allow a more direct translation between preclinical models of relapse in cocaine-addiction and the human condition being modeled.

In the present study, relapse to cocaine use within the first 30 days post-treatment was used to identify cocaine-addicted individuals at greatest risk of early relapse. Based on evidence linking the vmPFC/rACC to reduced CMA output and reduced cocaine-seeking (41, 44, 45), as well as enhanced vmPFC–amygdala coupling to reduced negative affect/reactivity to stress (41, 48, 49, 51), we expected relapse within the first 30 days post-treatment to be predicted by reduced connectivity between the CMA and vmPFC/rACC during treatment. In addition, drawing on evidence that coupling between the BLA and rodent homolog of the dmPFC/dACC facilitates the reinstatement of extinguished cocaine-seeking (42, 43), we hypothesized that enhanced BLA–dmPFC/dACC connectivity during treatment would also predict early relapse. We also explored amygdala circuitry at the whole-brain level to identify novel circuits that may arise as a function of relapse risk. Finally, we assessed for relapse-related differences in neurocognitive measures of executive control, emotional decision making and trait measures of stress reactivity previously associated with medial prefrontal to amygdala coupling (51, 63) as well as measures of cocaine craving and obsessive-compulsive cocaine use.

## Materials and Methods

### Subjects

Forty-five individuals, who met criteria for cocaine dependence on the Structured Clinical Interview for DSM-IV-TR axis I disorders (SCID-I), were recruited for the current study along with 22 healthy controls. Individuals were excluded if they had any history of major illness, were left-handed, had an estimated IQ below 70 [per the Wechsler test of adult reading (WTAR)], or met criteria for any neurological or active axis I disorder (other than substance use disorders) or were on psychotropic medications. Other drug use among cocaine-addicted subjects was not a condition for exclusion as long as cocaine dependence was the primary diagnosis.

All aspects of the research protocol were reviewed and approved by the Institutional Review Boards of the University of Texas Southwestern Medical Center at Dallas and the Veterans Administration North Texas Health Care System. Subjects provided informed consent prior to study participation.

### Procedure

Cocaine-addicted participants were recruited following admission to one of three residential cocaine dependence treatment programs: Veteran’s Administration Medical Center, Homeward Bound, Inc., or Nexus Recovery Center in Dallas, TX, USA. Each program utilized the Minnesota Model psychosocial treatment approach. Cocaine-addicted participants were admitted as soon as possible after last reported use of cocaine. Urine drug screens were conducted throughout residential treatment to verify abstinence. Control and cocaine-addicted participants completed a 5-min high resolution anatomical scan and a 6-min resting BOLD scan during which they were instructed to lie as still as possible with their eyes open. Cocaine-addicted individuals completed scans during their final week of treatment. All participants also completed personality, neurocognitive, and clinical measures within 1 week of their scan visit. Following discharge, follow-up sessions occurred twice weekly (once by phone), and included a structured interview assessing substance use since their previous visit and a urine drug screen. Relapse was defined as any use of cocaine or amphetamine since discharge and marked as the day of first use, or the day of their first missed appointment if participants missed two consecutive appointments. For analyses reported here, users were categorized as relapsed and non-relapsed based on their relapse status at day 30 following discharge from treatment.

### Measures

#### Trait measures of stress reactivity

Participants completed the temperament and character inventory (TCI) (64), and the NEO personality inventory (NEO-PI-R) (65). Here we focus on the TCI harm avoidance scale and the NEO Neuroticism scale as indices of trait reactivity to stress/anxiety. The TCI harm avoidance scale is a 36-item scale that assesses the propensity to worry excessively, fearfulness, shyness, and pessimism and propensity to easily fatigue (64). The NEO Neuroticism scale includes 48 items that assess the propensity to experience psychological distress and negative mood states such as anxiety, anger, hostility, fear, and depressed mood (65). Both scales have previously been associated with connectivity between the amygdala and medial prefrontal cortex (51, 63, 66).

#### Neurocognitive measures

The Wisconsin Card Sorting Test (WCST) (67) is used to assess executive control and is sensitive to dorsolateral, prefrontal, and orbitofrontal cortical function (68, 69). The task requires participants to sort a total of 128 cards (two sets of 64) on the basis of one of three principles: color, form, or number of symbols displayed on each card. Once 10 consecutive cards have been sorted according to a given principle, the principle shifts. The participant needs to notice the shift and learn to sort the cards according to the new sorting principle. In the present study, we were particularly interested in perseverative responding, the failure to change strategies following a principle shift. Cocaine-addicted individuals show deficits in perseverative responding on the WCST, even when other cognitive functions assessed by the task are otherwise preserved (70). In the present study, the total number of perseverative responses was natural log transformed to normalize the distribution prior to conducting group contrasts.

The Iowa Gambling Task (IGT) is designed to simulate real-life decisions involving uncertainty, reward, and punishment and is shown to be sensitive to vmPFC function as well as decision-making deficits seen in cocaine-addicted individuals (47). The IGT involves 100 choices from four decks of cards (A–D). Decks A and B yield a larger short-term payoff than decks C and D, but the accumulated penalties in decks A and B are larger than in decks C and D. Therefore, the optimal long-term strategy is to choose more cards from decks C and D. Subjects are instructed that the task was to accumulate as much (play) money as possible. In the current study, we assessed the total score on the IGT by dividing the total number of choices from decks C + D/A + B.

#### Measures of cocaine use characteristics

The Cocaine Craving Questionnaire (CCQ-Brief) (71) is a 10-item self-report measure adapted from the original 45-item CCQ-Now that asks subjects to indicate how much they agree or disagree with statements related to cocaine craving.

The Obsessive-Compulsive Cocaine use Scale (OCCS) is a 14-item self-report questionnaire adapted from the Obsessive-Compulsive Drinking Scale (OCDS) (72). It is being used to examine obsessive thoughts of cocaine use and compulsive cocaine use.

Cocaine use history was assessed with the Timeline Follow-Back (TLFB) (73). The TLFB uses a calendar and other memory aids to gather retrospective estimates of an individual’s daily substance use across the lifetime (from age of first use) as well as during the past 90 days. The TLFB also assesses the most money ever spent on cocaine in any 1 day, during one’s lifetime of use, and during the past 90 days of use.

### MRI data acquisition

Functional MRI data were collected on a 3-T Phillips MR scanner equipped with an eight channel RF coil. Thirty-six 3 mm thick functional slice locations were obtained in the axial plane parallel to the AC–PC line allowing for whole-brain coverage. Functional BOLD signals were acquired using a single-shot gradient-echo planar imaging (EPI) sequence with a matrix of 64 × 64, echo time (TE) of 25 ms, repetition time (TR) of 1.7 s, flip angle (FA) of 70°, field of view (FOV) of 208 mm × 208 mm yielding an in-plane resolution of 3.25 mm × 3.25 mm. For spatial normalization and localization, corresponding high resolution anatomical T1 images were acquired using 3D magnetization prepared rapid gradient-echo (MPRAGE) sequence with a TR/TE 8.2/3.8 ms, FA of 12°, and voxel size of 1 mm × 1 mm × 1 mm.

### Data processing

Data were preprocessed and analyzed using AFNI (74), FreeSurfer (http://surfer.nmr.mgh.harvard.edu/) and SPSS version 20. Following image reconstruction, resting data were submitted to slice-timing correction, motion correction, and quadratic detrending of time series data. A low band pass temporal filter was applied to restrict signal variation to frequencies between 0.01 and 0.1 Hz (54). Resting data images were then registered to standard (Talairach) space with a resampled resolution of 3.25 mm × 3.25 mm × 3 mm and smoothed with an isotropic 6 mm full-width half-maximum (FWHM) Gaussian kernel. To facilitate group analysis, an unbiased groupwise non-linear registration method was used to generate an implicit group reference image (75).

**Figure 1:**
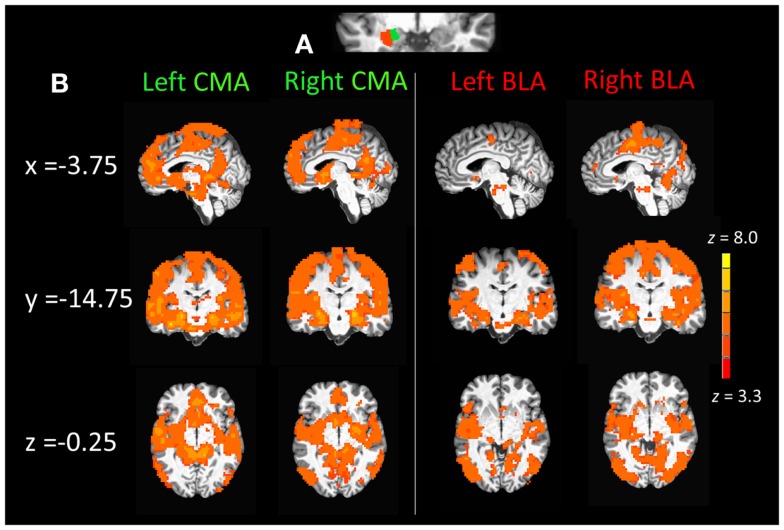
**(A)** Section of coronal anatomical slice (*y* = 1.75, Talairach standard space, 1 mm × 1 mm × 1 mm) with a single subjects basolateral amygdala (BLA) and corticomedial amygdala (CMA) division mask overlays (3 mm × 3 mm × 3mm) on the left amygdala. **(B)** Whole-brain resting state functional connectivity maps of the left and right CMA and BLA seeds for healthy control participants (*n* = 22), *p*_corrected_ = 0.01, *z*(21) > 3.3 and a cluster size of 55.

Probablistic maps derived from FSL’s Juelich Histological Atlas (76) were used to generate 50% probabilistic seed masks of the left and right BLA as well as left and right centromedial and superficial amygdala divisions, which were combined to create a CMA seed mask (16, 77). Individual left and right whole amygdala volumes were then generated using Freesurfer volumetric segmentation in original space. Segmentation and probabilistic volumes were then registered to standard (Talairach) space and combined to regenerate individual probabilistic seed volumes excluding voxels that did not fall within the segmentation amygdala volume (Figure 1A). Seed reference time courses within the left and right CMA and BLA seeds were regressed against the whole-brain to generate cross correlation (*CC*) maps. Importantly, seed time courses were extracted prior to applying the 6-mm FWHM spatial smoothing procedure described above. Time courses for six motion parameters and fluctuations in BOLD signals from cerebrospinal fluid and white matter were modeled as nuisance variables. Finally, *CC* distributions were normalized by applying Fisher’s *z*-transformation.

### Data analysis

Group differences in demographics, cocaine use characteristics, treatment characteristics, trait stress reactivity/anxiety, and neurocognitive measures were examined with one-way between-subjects ANOVAs, Chi-squared tests (for differences in frequencies) and Bonferroni corrected for three *post hoc* group contrasts (corrected threshold, *p* = 0.017). Only one previous study has assessed whole-brain connectivity for different amygdala subdivisions in a human sample (78). Thus prior to examining relapse-related differences in BLA/CMA connectivity, we were first interested in seeing what whole-brain connectivity with the BLA and CMA looks like in a healthy human brain. To address this, we generated whole-brain connectivity maps for the left and right CMA and BLA seeds for healthy controls alone. Connectivity maps were generated using one-sample *t*-tests (AFNIs 3dttest++) with a threshold of *t*(21) = 3.8 and cluster size of 55 voxels equivalent to a corrected *p* = 0.01.

To test our hypotheses that connectivity within BLA and CMA circuits would vary as a function of relapse risk, a general linear mixed model was run at the whole-brain level for each amygdala seed with group (relapse vs. non-relapse) entered as fixed effects. Where differences between relapse groups emerged, *post hoc* contrasts were conducted between each relapse group and the healthy control group to facilitate interpretation of the relapse-related group difference (i.e., which group is more like the healthy control group). Effects were considered significant if they passed an uncorrected voxel-wise threshold of *p* = 0.005 and corrected clusterwise threshold of *p* = 0.05. If relapse groups differed on a demographic characteristic, *post hoc* procedures were conducted to determine whether this characteristic could account for group effects observed in amygdala connectivity. Firstly, a bivariate correlation or independent samples *t*-test was conducted to examine mean connectivity in clusters where group effects emerged as a function of variance in this characteristic. If a significant association was observed, the whole-brain contrast was repeated including this characteristic as a covariate in the model to see if effects remained significant.

Additionally, where relapse groups differed in both resting connectivity and performance on neurocognitive measures, trait reactivity to stress, or clinical characteristics, bivariate correlations were conducted between amygdala connectivity within significant clusters (controlling for years of education) and individual variance on this measure/characteristic. All *post hoc* analyses were Bonferroni corrected based on the number of analyses conducted.

Finally, where differences in BLA/CMA connectivity emerged as a function of relapse risk, the effect size of these effects was estimated employing a leave-one-out cross-validation procedure proposed by Esterman and colleagues (79). For the first step, relapse vs. non-relapse contrasts for seeds where group effects emerged were re-run 45 times per seed, each time leaving a single person out of the contrast. For each of the 45 iterations average connectivity for the cluster showing the largest overlap with each of the full group contrast effects (see Figure 3) was extracted. During this procedure original group contrast effects were only used to identify clusters in the same brain regions, they were not employed as masks during the whole-brain contrasts or when extracting mean connectivity. Consequently, for each iteration, the clusters extracted were unique and independent of the individual left out of the analysis.

Average connectivity for each cluster was then entered into a single logistic regression model as predictors of relapse status. The logistic model generated was then used to predict the relapse status of the participant left out of that analysis run. Specificity, sensitivity, and overall model accuracy were reported. To examine the effect size of amygdala connectivity relative to other characteristics that may differentiate relapse from non-relapse participants, we generated additional logistic models including non-connectivity characteristics and connectivity effects as individual predictors in each model. A third set of models were tested including both connectivity and non-connectivity characteristics as predictors of relapse risk. All logistic regression models employed a classification cut-off of 0.5.

## Results

### Participant demographic and clinical characteristics

Twenty-four cocaine-addicted participants were classified as relapsed at 30-days post-treatment based on cocaine or psychostimulant drug use. The remaining 21 cocaine-addicted individuals were considered non-relapsed. As illustrated in Table 1, non-relapse individuals reported more years of education (*p* = 0.011) and fewer years smoking (*p* = 0.047) than relapse individuals, but did not differ on any other demographic characteristics. Importantly, no relapse-related group differences emerged for cocaine use history, self-reported craving or scores on the obsessive-compulsive cocaine use scale (all *p*s > 0.10). Non-relapse and relapse groups also did not differ in treatment duration or treatment center attended (all *p*s > 0.10) or other drug use. Table 1 also illustrates demographic and drug use characteristics of healthy control participants, highlighting where controls differed significantly from either relapse or non-relapse participants.

**Table 1:** **Demographic, drug use, treatment, and behavioral characteristics**.

Participant characteristic	Control (*n* = 22)	Non-relapsed at day 30 (*n* = 21)	Relapsed at day 30 (*n* = 24)
Age	42.05 (8.40)	43.10 (6.84)	43.75 (7.53)
Years of education^a^^,^^b^^,^**	13.91 (1.41)	13.29 (2.05)	11.83 (1.88)
IQ^b^^,^*	96.86 (10.26)	90.71 (9.35)	88.61 (8.63)
Gender (no. males)	14	18	21
Cigarette smokers^b^^,^^c^^,^** (no. smokers)	1	16	19
Cigarettes/day	–	12.94 (2.63)	16.00 (8.52)
Years smoking cigarettes^a^^,^*	–	17.88 (9.07)	24.32 (9.32)
Alcohol/week (no. standard drinks)^b^^,^^c^^,^**	2.06 (2.09)	2.76 (2.28)	2.50 (2.30)
Current opiate use (no. users)	0	1	2
Current stimulant use (no. users)	0	0	1
Current cannabis use (no. users)	0	3	3
Current other drug use (no. users)	0	1	0
Age of onset cocaine dependence	NA	27.15 (7.46)	26.48 (9.59)
Days cocaine used – last 90 days	0	71.43 (21.51)	69.88 (24.65)
Years cocaine used – lifetime	0	7.72 (3.99)	8.88 (6.48)
Days since last cocaine use	0	22.81 (4.31)	22.58 (3.62)
Amount spent on cocaine – last 90 days	0	$8075.05 (6296.51)	$5910.67 (5484.34)
Treatment center (no. in A–C)^d^	NA	3, 16, 2	7, 14, 3
Treatment duration (days)	NA	24.74 (8.08)	26.19 (14.04)
Craving – CCQ-brief	NA	19.48 (10.73)	18.39 (13.15)
OCCS compulsions	NA	14.81 (3.37)	13.05.33 (4.05)
OCCS obsessions	NA	10.19 (4.19)	9.27 (4.45)
NEO – neuroticism^b^^,^^c^^,^**	45.96 (9.94)	57.15 (9.18)	57.00 (10.49)
TCI – harm avoidance^b^^,^^c^^,^**	7.65 (4.20)	12.15 (6.23)	12.27 (4.69)
WCST – perseverative responses	14.27 (13.43)	17.21 (9.71)	24.79 (16.9)
IGT – total score	1.64 (26.38)	−3.36 (15.76)	−9.10 (11.57)

All values indicate mean with standard deviation in parentheses unless otherwise indicated. *p < 0.05, **p < 0.01 for group main effects.

*^a^ Difference between relapse and non-relapse*.

*^b^ Difference between relapse and controls*.

*^c^ Difference between non-relapse and controls*.

*A, Veteran’s Administration Medical Center; B, Homeward Bound, Inc.; C, Nexus Recovery Center*.

*^d^ Treatment centers based in Dallas, TX, USA*.

*Cocaine-addicted participants had been abstinent from all drugs of abuse for a minimum of 17 days at the day of scanning*.

*Note: A portion of Table 1 was previously reported in McHugh and colleagues (103)*.

### Neurocognitive performance and trait stress reactivity

Performance on neurocognitive and trait stress reactivity/anxiety measures is summarized in Table 1. No difference between relapse and non-relapse individuals emerged for total IGT score, perseverative responding on the WCST, trait Harm Avoidance, or Neuroticism (all *p*s > 20), even after controlling for differences in years of education (all *p*s > 0.26). Since performance and scores across these measures did not differ between the two cocaine groups, their contribution to relapse-related connectivity effects was not further considered.

Compared to healthy control participants, relapse participants evidenced more perseverative responding on the WCST (*p* = 0.009), but this difference was no longer significant after controlling for differences in IQ (*p* = 0.104). Healthy controls scored significantly lower on Neuroticism and Harm Avoidance than both relapse (*p* = 0.001 and *p* = 0.012) and non-relapse individuals (*p* = 0.001 and *p* = 0.015).

### Amygdala connectivity – healthy controls

#### Corticomedial amygdala connectivity

Figure 1B displays mean whole-brain connectivity in healthy control participants for the left and right CMA seeds, which revealed similar patterns of whole-brain connectivity. Positive connectivity was seen in the medial prefrontal cortex extending from the vmPFC up into the anterior dmPFC and also encompassing the entire extent of the rACC from subgenual into pregenual ACC. Positive connectivity was also seen in a more posterior dmPFC cluster, which extended laterally into pre- and post-central gyri and superior and middle temporal gyri. The left and right CMA also showed significant connectivity with the posterior cingulate cortex and the precuneus, as well as the parahippocampal gyrus, extending into the anterior insula and inferior frontal gyrus, along with the striatum, specifically the caudate and nucleus accumbens.

#### Basolateral amygdala connectivity

As illustrated in the right panel of Figure 1B, connectivity with the right and left BLA seeds emerged in middle and superior temporal cortical regions. Positive connectivity was also seen in posterior cortical regions including the superior parietal cortex, cuneus, lingual, and fusiform gyri as well as the inferior and middle occipital gyri. The left and right BLA seeds also displayed connectivity with the posterior thalamus as well as the posterior dmPFC extending into pre- and post-central gyri as well as the posterior insula extending into the middle temporal gyrus.

### Amygdala connectivity and relapse risk

#### Corticomedial amygdala circuits

**Figure 2:**
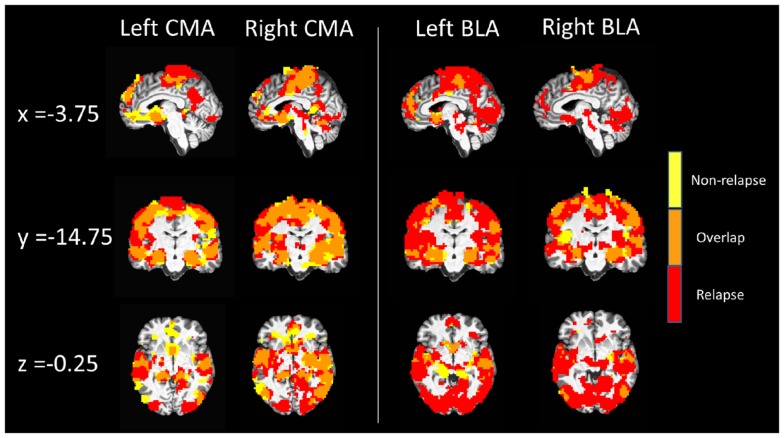
**Whole-brain resting state functional connectivity maps of left and right CMA and BLA seeds for relapse (*n* = 24, red) and non-relapse (*n* = 21, yellow) groups**. Maps are overlaid for display purposes, with regions where whole-brain connectivity group maps overlapped displayed in orange, *p*_corrected_ = 0.01, *z*(21) > 3.3 and a cluster size of 55.

**Figure 3:**
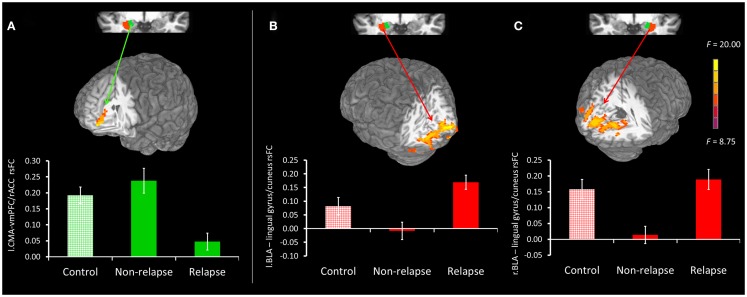
**(A)** Top panel presents non-relapse vs. relapse whole-brain contrast for the left CMA (green), showing regions where non-relapse individuals evidenced significantly greater rsFC than relapse individuals. Bottom panel reflects mean connectivity within the displayed cluster for controls, non-relapse and relapse. **(B,C)** Top panel presents relapse vs. non-relapse whole-brain contrast for the left BLA (red) and right BLA (red) respectively. Clusters show regions where relapse individuals evidenced significantly greater rsFC than non-relapse individuals. The bottom panel reflects the mean connectivity within these clusters for controls, relapse and non-relapse individuals. Mean connectivity for healthy controls is presented for display purposes only. Whole-brain contrast effects are presented at *p*_uncorrected_ = 0.005 and clusterwise thresholded at *p*_corrected_ = 0.05. Error bars reflect SEM.

Figure 2 displays maps of whole-brain connectivity for left and right CMA seeds illustrating regions of unique and overlapping connectivity for relapse and non-relapse groups. Regions where significant differences between these two groups emerged are further displayed in the contrast presented in Figure 3A. As illustrated, relapse individuals evidenced significantly reduced connectivity between the left CMA and a cluster within the vmPFC extending into the rACC (Figure 3A; Table 2). As indicated in Figure 3A, mean connectivity within this vmPFC/rACC cluster is similar for non-relapse and control participants, suggesting that effects do indeed reflect reduced connectivity in relapse participants, not enhanced connectivity in non-relapse participants. Whole-brain contrasts conducted between control and relapse participants revealed a cluster which overlapped with relapse vs. non-relapse contrast effect displayed in Figure 3A. Relapse individuals displayed significantly reduced connectivity relative to controls within this cluster (see Table 2). No significant group effects emerged for the right CMA seed. In cocaine-addicted individuals, connectivity between the vmPFC and CMA was also unrelated to years of education (*p* = 0.750) and years smoking cigarettes (*p* = 0.146) confirming that relapse-related connectivity differences could not be attributed to group differences on these two pre-treatment characteristics.

**Table 2:** **Summary of significant clusters arising from whole-brain group contrasts**.

Resting connectivity contrast (seed to cluster)	Cluster size (# voxels)	Peak differences *F* value	Peak coordinates (Talairach)		
			*L*	*P*	*I*
**LEFT CMA To vmPFC/rACC**					
Non-relapse vs. relapse	70	17.48	7.5	46.5	−12.5
Controls vs. relapse	195	7.5	7.5	49.5	+17.5
**LEFT BLA TO LINGUAL GYRUS/CUNEUS**					
Relapse vs. non-relapse	582	20.07	16.5	−85.5	−0.5
**RIGHT BLA TO LINGUAL GYRUS/CUNEUS**					
Relapse vs. non-relapse	1131	17.77	−13.5	−73.5	−12.5
Controls vs. non-relapse	304	20.47	13.5	−64.5	−0.5

*Whole-brain contrast effects are presented at *p*_uncorrected_ = 0.005 and clusterwise thresholded at *p*_corrected_ = 0.05. *Post hoc* bivariate correlations confirmed that relapse-related differences in BLA and CMA connectivity were not related to years of smoking or years of education (all *p*s > 0.17). L = Left-to-Right, P = Posterior-to-Anterior, I = Inferior-to-Superior*.

#### Basolateral amygdala seed

Whole-brain connectivity for the left and right BLA seeds for non-relapse and relapse groups are displayed in the right hand panel of Figure 2. Regions where the two groups significantly differed are illustrated in Figures 3B,C. Non-relapse participants evidenced significantly reduced connectivity between the right BLA seed and a large cluster encompassing the cuneus and lingual guyrus, extending into the parahippocampal gyrus (Figure 3C; Table 2). Non-relapse individuals also evidenced reduced connectivity between the left BLA and a similar but smaller cluster encompassing the cuneus and lingual gyrus relative to relapse participants (Figure 3B; Table 2). Non-relapse participants also evidenced reduced connectivity between the left BLA and a cluster in the middle temporal gyrus. However, connectivity within this cluster in cocaine-addicted individuals was negatively correlated with years of education, *r*(44) = −0.301, *p* = 0.047, and when we re-ran the whole-brain contrast including years of education as a covariate, this effect disappeared. In contrast, connectivity between the left BLA and lingual gyrus/cuneus was unrelated to years of education (*p* = 0.359) or years smoking cigarettes (*p* = 0.525).

Finally, as illustrated in the bottom panel of Figures 3B,C, mean connectivity of healthy control participants most closely resembled that of relapse individuals for both visual cortical clusters. Non-relapse vs. controls whole-brain contrasts revealed significantly reduced connectivity between the right BLA and an overlapping cluster within the left lingual gyrus in non-relapse participants (Table 2).

#### Cross-validation analysis

Table 3 illustrates the results of seven leave-one-out cross-validation models conducted to estimate the effect size of years of education, years smoking cigarettes, and amygdala connectivity as markers of early relapse risk. Models 1–5 examine each of the five predictors (years of education, years smoking cigarettes, CMA–vmPFC/rACC, left BLA-lingual gyrus/cuneus, and right BLA-lingual gyrus/cuneus) alone as predictors of relapse by day 30. Years smoking cigarettes (Model 1) did not perform significantly above chance (*p* = 0.203). By contrast, the remaining four models performed marginally above chance (all *p*s = 0.06) and demonstrate equivalent accuracy. Years of education shows the strongest sensitivity as an individual predictor, correctly classifying 18 of 24 relapsers, but poorest specificity, incorrectly classifying 10 of 21 non-relapsers.

**Table 3:** **Summary of leave-one-out cross-validation models predicting relapse status at day 30 post-treatment based on rsFC, years smoking cigarettes, and years of education**.

Cross-validation models	Sensitivity (%)	Specificity (%)	Accuracy (%)	χ^2^	Sig (*p* value)
Model 1
Years smoking cigarettes	70.8	47.6	60.0	1.622	0.203
Model 2
Years of education alone	75.0	52.4	64.4	3.57	0.06
Model 3
Left CMA–vmPFC/rACC	66.7	61.9	64.4	3.67	0.06
Model 4
Right BLA-lingual gyrus/cuneus	70.8	57.1	64.4	3.59	0.06
Model 5
Left BLA-lingual gyrus/cuneus	66.7	61.9	64.4	3.67	0.06
Model 6
Left CMA–vmPFC/rACC	79.2	66.7	73.3	9.64	0.002
Right BLA-lingual gyrus/cuneus	
Left BLA-lingual gyrus/cuneus	
Model 7
Years of Education	70.8	76.2	73.3	9.91	0.002
Right CMA–vmPFC/rACC	
Right BLA-lingual gyrus/cuneus	
Left BLA-lingual gyrus/cuneus	

The three rsFC predictors all show similar sensitivity, specificity, and accuracy. Model 6 examines the three rsFC predictors in a single model. This model performed significantly above chance (*p* = 0.002). Relative to Models 1–5, Model 6 showed both improved sensitivity, 19 of 24 relapsers correctly classified, and specificity, 14 of 21 non-relapsers correctly classified. Finally, we tested a model including years of education and rsFC as predictors of relapse (Model 7). Relative to Model 6, this model showed poorer sensitivity, 17 of 24 relapsers correctly classified, but improved specificity, 16 of 21 non-relapsers correctly classified. Model 6 also performed significantly above chance (*p* = 0.002) in predicting 30-day relapse status.

## Discussion

The current study aimed to address the contribution of BLA and CMA circuits to relapse risk in cocaine-addicted individuals. Drawing from preclinical models implicating distinct roles of the two divisions in relapse to cocaine use, as well as human, non-human primate, and rodent studies of prefrontal-amygdala circuitry, it was expected that early relapse risk would be associated with reduced CMA–vmPFC/rACC connectivity and enhanced BLA–dmPFC/dACC connectivity. The role of additional amygdala circuitry in early relapse risk was also explored. As expected, individuals who relapsed to cocaine use within the first 30 days post-treatment displayed reduced rsFC between the left CMA and vmPFC/rACC. This finding extends on earlier work by our group showing reduced rsFC between the amygdala and vmPFC in current cocaine users (62). No group differences emerged in resting connectivity between the dmPFC/dACC and the BLA. Instead, individuals who remained abstinent up to 30 days post-treatment evidenced a robust reduction in connectivity between the bilateral BLA and visual cortex (lingual gyrus/cuneus).

Individuals who relapsed early also evidenced fewer years of education, but this effect was independent of the connectivity effects just described. A series of cross-validation analyses indicated that years of education and rsFC within each of the above three circuits perform similarly as individual markers of relapse risk. Model accuracy, sensitivity, and specificity significantly improved when rsFC variables were considered together in a single model. The greatest sensitivity to relapse risk was observed for a model including rsFC in all three circuits. Including years of education improved the specificity of this model, but at the expense of model sensitivity.

Before discussing these effects further, it should be mentioned that the distribution of whole-brain connectivity seen for the CMA and BLA in healthy controls in the present study is largely consistent with that reported by Roy and colleagues (78). However, some differences between the two sets of findings were present. In particular, Roy and colleagues reported patterns of both significant positive and negative connectivity with amygdala divisions where as we observed only significant positive connectivity. This difference in direction most likely reflects a processing step adopted by Roy and colleagues involving removal of global variations in the BOLD signal (global signal regression) prior to generating rsFC maps. This method has been shown to potentially introduce spurious negative correlations into rsFC maps (80) and for this reason was not used in the present study. Although the present study did not set out to directly address the dissociability of CMA and BLA circuits, the findings do suggest a functional dissociation. Specifically, relapse-related effects were present between the CMA and vmPFC/rACC but not between the BLA and vmPFC/rACC, and vice versa for the lingual gyrus/cuneus. Moreover, the functional specificity of relapse-related alterations in rsFC between the CMA and vmPFC/rACC is consistent with preclinical evidence that the vmPFC down regulates amygdala output via indirect projections to the CMA, not the BLA (41).

### Early relapse risk is associated with reduced connectivity between the CMA and vmPFC

The CMA, in particular the CeA, has been implicated in the incubation of cocaine craving as well as facilitating stress-induced reinstatement and withdrawal-induced negative affect and drug-seeking in preclinical models of addiction (28, 29, 33–35, 81). In contrast, the vmPFC exerts an inhibitory effect on drug-seeking behavior, facilitating extinction as well as inhibiting reinstatement of cocaine-seeking (41, 44, 45, 82). For example, a recent study demonstrated that deficits in the vmPFC of rats with an extended history of cocaine self-administration results in greater resistance to the extinction of cocaine-seeking (82).

Findings from human and animal studies suggest that vmPFC–amygdala circuits function to down-regulate amygdala output (41, 48–53, 83), facilitating the regulation of affective responses mediated by the amygdala (50, 83). In humans, enhanced structural integrity of the fiber tract connecting the vmPFC/rACC to the amygdala (51), as well as greater functional connectivity between the vmPFC/rACC and amgydala (66), is associated with reduced trait reactivity to stress/trait anxiety. Amygdala–vmPFC/rACC coupling has also been linked to state-dependent changes in reactivity to aversive stimuli and negative mood (84, 85). In the present study, relapse-related differences in CMA–vmPFC/rACC connectivity were not accompanied by relapse vs. non-relapse differences in self-reported Neuroticism or Harm Avoidance. While this suggests that group differences in CMA–vmPFC/rACC connectivity do not reflect trait variability in anxiety/stress reactivity, it does not rule out the possibility that rsFC in this circuit varies as a function of abstinence-related changes in reactivity to stress/anxiety previously linked to the CMA (9, 33). Unfortunately, measures sensitive to state changes in mood/reactivity to stress were not administered in the current study, limiting our capacity to draw such inferences.

In addition to the possible mediation of abstinence-related changes in stress reactivity/anxiety, preclinical evidence suggests that reduced vmPFC/rACC input to the CMA may directly facilitate the reinstatement of cocaine-seeking. Stimulation of the vmPFC in rodents reduces responsiveness of the CeA of the CMA division to excitatory inputs from the BLA and insula cortex (53). The CeA sends excitatory glutamatergic projections to the ventral tegmental area, which in turn sends dopaminergic input to the nucleus accumbens and dmPFC, a process implicated in the reinstatement of cocaine-seeking (36). Thus reduced vmPFC input to the CMA may have a disinhibitory effect on CMA output, resulting in activation of a pathway known to mediate the reinstatement of cocaine-seeking. While rsFC in the current study cannot be used to infer directionality or causality, the mechanism outlined presents a plausible basis through which reduced vmPFC-CMA connectivity may confer relapse risk. Future studies in both humans and animals are required to further explore and validate such a mechanism.

### Early relapse risk is associated with connectivity between the BLA and occipital cortex

The BLA receives extensive input from the visual cortex and other sensory regions (16, 17). These projections support the acquisition and evaluation of emotional/motivational salience of a given sensory stimuli (16). The BLA also sends direct (monosynaptic) projections back to the ventral visual stream (86). These projections are implicated in top-down regulation mechanisms that modulate visual processing of emotional stimuli (86, 87). Presentation of emotionally/motivationally salient stimuli (e.g., fearful faces/drug cues) produces robust activation in both the amygdala and visual cortex, as well as enhanced amygdala–visual cortical coupling relative to neutral stimuli (87–91). Critically, differences in visual cortical activation to emotional vs. neutral stimuli are not present in individuals with amygdala damage (92), supporting the view that projections from the amygdala to the visual cortex mediate differential sensory processing of emotionally salient vs. neutral stimuli.

Reorganization of amygdala projections to the visual cortex has been implicated as a basis for treatment success following exposure therapy for specific phobias, potentially by regulating the sensory processing of fear-relevant stimuli (93). Reduced connectivity between the BLA and the ventral and caudal visual cortex in the present study may reflect a similar mechanism in relation to top-down processing of drug cues and stressful environmental stimuli that may otherwise precipitate early relapse in cocaine-addicted individuals. While speculative, this mechanism accords with evidence that long-term abstinence from drugs of abuse (89) as well as cognitive regulation of craving (94) both attenuate differential engagement of the visual cortex to drug-related vs. neutral visual cues. In sum, the robust suppression of BLA–visual cortical coupling seen among non-relapse individuals is a novel finding but consistent with the putative top-down regulatory role of re-entrant projections from the BLA to the visual cortex. Future studies are needed to replicate this finding and examine the extent to which variance in BLA–visual cortical coupling at rest mediates craving and visual cortical activation to drug-related or stress/anxiety invoking visual cues. It will also be of interest to see whether relapse risk varies as a function of connectivity between the BLA and visual cortical regions during the presentation of stress or drug-related cues.

### Implications for treatment

Preclinical models of cocaine addiction have identified the BLA and CMA as key players in relapse following forced abstinence or extinction of cocaine-seeking. In the present study, we seeded these amygdala subregions and show that resting connectivity measured prior to discharge can be utilized to identify individuals at greatest risk of early relapse. By contrast, early relapse risk was neither related to pre-treatment clinical characteristics such as craving, amount of recent cocaine use or years of use, nor to performance across behavioral measures of executive control or trait measures of stress reactivity/anxiety. In fact, years of education was the only pre-treatment characteristics that varied significantly between our two cocaine groups. While the carefully selected sample may not mirror the patient typically presenting for treatment, our inclusion/exclusion criteria allowed us to parse out the neural disruptions relevant to cocaine relapse without the potential confounds of recent substance use, associated psychiatric disorders or psychotropic medications. Although the current findings need replication in an independent sample, they highlight the potential utility of resting connectivity as a tool for probing vulnerability to relapse during (or potentially prior to) treatment for cocaine addiction.

Functional coupling within the cortico-amygdala circuits identified here as markers of early relapse risk may also present novel treatment targets. Models of cognitive affect regulation posit that the vmPFC acts as a central mediator between cognitive control and executive attention regions, such as the dorsolateral PFC (dlPFC) and posterior parietal cortex, which have otherwise sparse connections to emotion generation regions such as the amygdala (95). Accordingly, in the present study, we did not observe significant connectivity between our amygdala seeds and either the dlPFC or posterior parietal cortex. However, we would posit that cognitive strategies, such as working memory training (96), which enhance engagement of cognitive control regions like the dlPFC could produce sustained alterations in functional coupling between the vmPFC and amygdala. As an example, reappraisal is a cognitive affect regulation strategy known to engage the dlPFC (97). The dlPFC in turn recruits the vmPFC which projects to and modulates amygdala output (95). Future studies should assess whether downstream consequences of improved recruitment of executive control regions include affect/craving regulation and amygdala–vmPFC coupling.

It is also possible that reduced CMA–vmPFC/rACC coupling among individuals who relapsed early to cocaine use simply reflects diminished cognitive resources. A recent study showed that employing methods to acutely attenuate available cognitive resources produced a significant reduction in positive coupling between the vmPFC and amygdala as well as enhanced amygdala activation to negative emotional scenes (85). Conversely, it may be possible to rescue positive coupling between the vmPFC and amygdala by introducing interventions that produce a general enhancement in available cognitive resources. Pharmacotherapies that enhance cognitive function (e.g., modafinal, methylphenidate) have been tried as treatments for stimulant addiction with mixed success (98, 99). It may be that resting connectivity between the CMA and vmPFC could be utilized to identify individuals who would be most responsive to treatment with cognitive enhancers. This view finds some support from a recent study that identified the vmPFC as the primary neural candidate underlying improved inhibitory control in cocaine-dependent individuals treated with methylphenidate (100). Here, cocaine-addicted individuals showing the greatest improvement in inhibitory control also showed the largest change in vmPFC activation during response inhibition under methylphenidate.

Another potential intervention for promoting clinically significant changes in vmPFC–amygdala coupling is repetitive transcranial magnetic stimulation (rTMS). A recent study found that low frequency rTMS delivered to the right dlPFC for the treatment of depression produced enhanced resting cerebral blood flow to the vmPFC (101). Critically, it was the latter effect, not cerebral blood flow to the dlPFC that predicted positive treatment outcomes. Importantly, this finding is consistent with the emotion-regulation model discussed above, whereby the dlPFC indirectly affects limbic regions such as the amygdala by altering functioning within the vmPFC (95). Treatment with rTMS to the dlPFC has also been found to produce significant reductions in self-reported craving among cocaine-addicted individuals (102). While these findings are promising, future studies that couple rTMS to the dlPFC with measures of resting connectivity between the CMA and vmPFC/rACC as well as concurrent measures of affect and craving are needed. Moreover, if through replication CMA–vmPFC/rACC coupling turns out to be a reliable marker of relapse risk, resting connectivity within this circuit could potentially be used to individually titrate the amount of rTMS or individually adjust rTMS parameters to produce clinically significant change.

### Limitations and concluding remarks

Some limitations of the present study should be noted. Firstly, it is important to stress that rsFC effects cannot be used to infer directionality or causality (e.g., vmPFC down-regulating amygdala output) within the circuits identified here as potential neural candidates for relapse risk. Secondly, although clinical, neurocognitive and trait measures did not predict early relapse and thus could not account for relapse-related differences in rsFC, it is possible that connectivity effects reflect abstinence-related changes in affect and behavior not measured in the present study. To avoid this limitation and facilitate interpretation of connectivity effects, it is important that future studies administer contemporaneous measures of state anxiety/stress reactivity and cue reactivity on the day of the scanning. Moreover, it will be of interest to see whether these relapse-related connectivity effects at rest are also seen during manipulations designed to probe these circuits, such as drug cue or stress reactivity paradigms. Despite these limitations, the present study demonstrated that by capitalizing on preclinical models of relapse to cocaine use and rsFC as a tool for probing neural circuits, we can identify promising neural markers of early relapse in cocaine-addicted individuals. Future efforts to replicate the current findings and alter connectivity within these circuits may yield novel interventions and facilitate treatment outcomes by identifying individuals at greatest risk of early relapse.

## Conflict of Interest Statement

The authors declare that the research was conducted in the absence of any commercial or financial relationships that could be construed as a potential conflict of interest.
